# No influence of CO_2_ on stable isotope analyses of soil waters with off‐axis integrated cavity output spectroscopy (OA‐ICOS)

**DOI:** 10.1002/rcm.7815

**Published:** 2017-02-05

**Authors:** Matthias Sprenger, Doerthe Tetzlaff, Chris Soulsby

**Affiliations:** ^1^ Northern Rivers Institute, School of GeosciencesUniversity of AberdeenElphinstone RoadAberdeenAB24 3UFUK

## Abstract

**Rationale:**

It was recently shown that the presence of CO_2_ affects the stable isotope (δ^2^H and δ^18^O values) analysis of water vapor via Wavelength‐Scanned Cavity Ring‐Down Spectroscopy. Here, we test how much CO_2_ is emitted from soil samples and if the CO_2_ in the headspace influences the isotope analysis with the direct equilibration method by Off‐Axis Integrated Cavity Output Spectroscopy (OA‐ICOS).

**Methods:**

The headspace above different amounts of sparkling water was sampled, and its stable isotopic composition (δ^2^H and δ^18^O values) and CO_2_ concentration were measured by direct equilibration and by gas chromatography, respectively. In addition, the headspace above soil samples was analyzed in the same way. Furthermore, the gravimetric water content and the loss on ignition were measured for the soil samples.

**Results:**

The experiment with the sparkling water showed that CO_2_ does not influence the stable isotope analysis by OA‐ICOS. CO_2_ was emitted from the soil samples and correlated with the isotopic fractionation signal, but no causal relationship between the two was determined. Instead, the fractionation signal in pore water isotopes can be explained by soil evaporation and the CO_2_ can be related to soil moisture and organic matter which both enhance microbial activity.

**Conclusions:**

We found, despite the high CO_2_ emissions from soil samples, no need for a post‐correction of the pore water stable isotope analysis results, since there is no relation between CO_2_ concentrations and the stable isotope results of vapor samples obtained with OA‐ICOS. © 2016 The Authors. Rapid Communications in Mass Spectrometry Published by John Wiley & Sons Ltd.

Stable isotope analysis of soil pore waters (δ^2^H and δ^18^O values) is increasingly being applied in various studies dealing with, for example, flow paths on hillslopes,^1^, ^2^ travel times in the vadose zone,^3^, ^4^ and root water uptake.^5^, ^6^ Several methods for the analysis of the stable isotopes of soil pore waters exist,^7^ but differences in their results are not yet fully understood.^7^, ^8^ Of the available methods, the application of the direct equilibration for stable isotope analysis of pore waters^9^ is increasingly used.^2^, ^3^, ^4^, ^10^, ^11^, ^12^ However, because this direct equilibration method uses laser spectrometry to determine the isotopic composition of the headspace in equilibrium with the soil pore water, volatile compounds can potentially alter the analysis by spectral interferences, when they absorb the laser in similar wavelengths to the isotopologues of water.^13^, ^14^

Such a spectral interference was recently found for CO_2_ during isotope analyses with the laser‐based technique of wavelength‐scanned cavity ring‐down spectrometry (WS‐CRDS):^15^ there is an apparent linear relationship between the offset of the isotopic analysis of a standard water vapor and the amount of CO_2_ in the vapor. While the δ^18^O values were overestimated with higher CO_2_ concentration, the δ^2^H values were underestimated.^15^ This offset induced by CO_2_, resulting in increased δ^2^H values and reduced δ^18^O values, would directly affect estimates of the so‐called deuterium excess (d‐excess).^16^

The d‐excess was introduced by Dansgaard^16^ as an index for non‐equilibrium conditions and is defined as:

(1) $${d‐\text{excess}=\delta }^{2}{H–8\times \delta }^{18}O$$

The isotopic signal of precipitation has a global average d‐excess of about 10 ‰, which results from equilibrium Rayleigh condensation from vapor that was evaporated in a non‐equilibrium process from seawater.^16^ When soil water that originated from precipitation (d‐excess = 10 ‰) evaporates, the residual water in the soil will have a d‐excess <10 ‰ due to non‐equilibrium processes (kinetic fractionation) and the δ^18^O‐ δ^2^H relationship of the water will have a slope <8 in the dual isotope space (the regression line is then called the evaporation line).^17^ Thus, the offset from the real isotope value induced by CO_2_ could be misinterpreted as a signal for evaporation of soil water. Since the δ^18^O‐δ^2^H relationship in precipitation varies locally, depending on various factors (e.g., temperature and humidity during cloud generation and condensation, altitude effects, continental effects, latitude effects),^18^ we use the line‐conditioned excess (lc‐excess) defined as:^19^

(2) $${\mathrm{lc}‐\text{excess}=\delta }^{2}{H–a\times \delta }^{18}O–b$$

where a and b represent the slope and intercept of the local meteoric water line (LMWL). For precipitation, the lc‐excess has an average value of 0 ‰ and soil water plotting below the LMWL will be of lc‐excess <0.

So far, the effect of CO_2_ on stable isotope analysis has only been tested for WS‐CRDS^15^ and there is a need to assess if or how CO_2_ also affects the analysis with Off‐Axis Integrated Cavity Output Spectroscopy (OA‐ICOS). Both analyzer systems use the absorption of a near‐infrared laser beam by molecules (i.e. isotopologues) in a gaseous sample in a high‐finesse optical cavity.^20^, ^21^ However, directing the laser beam off‐axis allows spatial separation of the multiple reflections within the cavity,^22^ which results in fully resolved OA‐ICOS absorption spectra.^23^ Therefore, unlike WS‐CRDS, OA‐ICOS does not derive the isotope ratios in the cavity from discrete wavelength sampling measurements. Instead, the isotope ratios are derived from the integrated areas under fully resolved absorption spectra (D. S. Baer, Los Gatos Research, personal communication). How this different instrumental set up for OA‐ICOS may affect the influence of CO_2_ on the measurements of stable isotopes of water vapor has not yet been studied.

As microbial activity is known to respond to temperature changes within a few hours^24^ and the aeration of the soil resulting from taking disturbed soil samples enhances soil respiration,^25^ it is likely that CO_2_ is emitted from the soil samples taken into the laboratory. This will probably be of special relevance if soils contain high levels of organic matter, as aeration and temperature increase have been shown to stimulate CO_2_ production in peaty soils.^26^ To date, it has not been tested whether CO_2_ is emitted while soil samples are stored prior to isotope analysis.

We address the two following research questions in this study. (1) Does CO_2_ alter stable isotope analysis by the direct equilibration method? (2) Do soil samples emit CO_2_ during storage in the laboratory?

## Experimental

### Set up

The experimental set up follows two tracks: First, we determined the isotopic composition of different volumes of sparkling water that were de‐gassing different amounts of CO_2_ to assess the influence of CO_2_ on the isotope analysis (Sparkling water experiment). Secondly, we conducted analysis of the pore water stable isotopes of field‐moist soil samples and measured the CO_2_ emissions from the samples in the lab (Soil water experiment).

**Figure 1:**
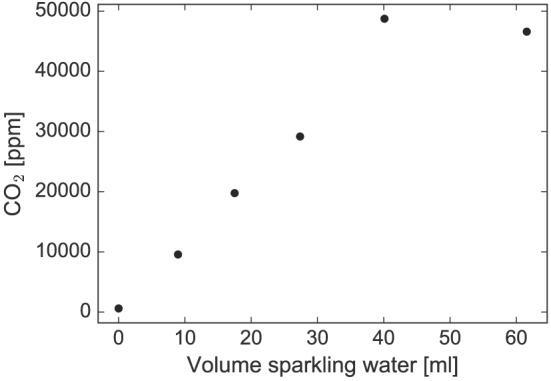
CO_2_ concentration in the headspace above different volumes of sparkling water after 2 days of storage.

For the sparkling water experiment, five samples of sparkling water with known isotopic composition (measured in liquid mode: δ^18^O = –7.45 ± 0.1 ‰ and δ^2^H = –50.8 ± 0.4 ‰, *n* = 3), but different volumes of sparkling water, were prepared in airtight bags (Weber Packaging, Güglingen, Germany). Another sample was prepared with non‐sparkling (still) water from the same spring having the same isotopic composition (measured in liquid mode: δ^18^O = –7.43 ± 0.1 ‰ and δ^2^H = –50.7 ± 0.4 ‰, *n* = 3). The isotopic composition of the water was determined by Off‐Axis Integrated Cavity Output Spectroscopy (OA‐ICOS) (triple water‐vapor isotope analyzer TWIA‐45‐EP, Model#: 912‐0032‐0000, Serial#: 14‐0038, Manufactured: 03/2014, Los Gatos Research, Inc., San Jose, CA, USA) running in liquid mode with a precision of ±0.4 ‰ for δ^2^H values and ±0.1 ‰ for δ^18^O values, as given by the manufacturer. The bags with the sparkling water were inflated with pressurized dry air before immediately then heat‐sealing them. The pressurized dry air had a CO_2_ concentration of about 600 ppm. The bags were stored for 2 days under constant temperature to allow for an equilibration between the water and the headspace in the bag until the stable isotope analysis were conducted as described below. The different water volumes of sparkling water exhausted different amounts of CO_2_ during the 2 days of storage before isotope and CO_2_ analyses (Table 1, Fig. 1).

**Table 1:** Sample characteristics of the sparkling water experiment with the volume of sample, the isotopic composition (δ^18^O and δ^2^H values) of the equilibrated headspace above the sparkling water samples and the CO_2_ concentration measured in the headspace

Sample ID	Non‐sparkling water [mL]	Sparkling water [mL]	δ^18^O [‰]	δ^2^H [‰]	CO_2_ [ppm]
δ_nsw_	22.7	0	‐7.5	‐50.8	600
δ_sw1_	0	9.0	‐7.5	‐50.2	9,563
δ_sw2_	0	17.5	‐7.2	‐49.4	19,756
δ_sw3_	0	27.4	‐7.3	‐50.1	29,163
δ_sw4_	0	40.1	‐7.1	‐50.2	48,726
δ_sw5_	0	61.6	‐7.4	‐50.7	46,578

To compare the isotope data from the non‐sparkling water (δ_nsw_ values) with those for the sparkling water (δ_sw_ values), we calculated the differences as Δδ = δ_sw_ – δ_nsw_.

For the soil water experiment, 24 soil samples were taken from peaty podzols of the Bruntland Burn experimental catchment in the Scottish Highlands. A detailed description of the hydrometric and isotopic dynamics of the study site was published previously.^27^ The soil sampling took place within the upper 20 cm of the profile, which is characterized by high organic matter content (17–80 %)^28^ and low bulk density (0.76 ± 0.21 g cm^–3^).^28^ We sampled depth profiles at eight different locations with each four samples taken in 5 cm increments (Table 2). Each sample consisted of 100–300 g of field‐wet soil that was stored in airtight bags (Weber Packaging) until performance of the isotope analysis in the lab. When sampling the soil in the field, it was ensured that as little air as possible was present in the bags with the soil samples by manually furling them (no vacuum applied). In this way, the isotopic equilibration and CO_2_ exhaust from the soil were limited to the 2 days of controlled storage after adding dry air in the laboratory. Thus, the CO_2_ measured after the isotope analysis would stem from the 2 days of storage prior to the analysis. The isotope analyses described below were performed within 1 week after soil sampling.

**Table 2:** Characterization of the soil samples, sampling depth, stable isotopic composition of the pore waters (δ^18^O and δ^2^H values), CO_2_ concentration in the headspace during the isotope analysis, gravimetric water content (GWC) loss on ignition (LOI) of the soil samples, and sampling date

Sample no.	Depth [cm]	δ^18^O [‰]	δ^2^H [‰]	CO_2_ [ppm]	GWC [‐]	LOI [%]	Sampling date
1	0–5	−6.0	−49.3	45,295	0.68	94	04.08.2016
2	5–10	−5.9	−48.4	49,917	0.70	81	04.08.2016
3	10–15	−6.1	−48.0	31,811	0.57	67	04.08.2016
4	15–20	−7.8	−54.1	10,790	0.33	29	04.08.2016
5	0–5	−6.5	−52.1	32,962	0.65	6	04.08.2016
6	5–10	−6.8	−49.7	33,597	0.28	9	04.08.2016
7	10–15	−7.5	−54.1	18,159	0.26	10	04.08.2016
8	15–20	−6.9	−52.4	10,434	0.32	24	04.08.2016
9	0–5	−6.2	−50.8	44,044	0.49	16	04.08.2016
10	5–10	−6.3	−48.5	19,077	0.31	9	04.08.2016
11	10–15	−7.3	−52.7	23,404	0.29	11	04.08.2016
12	15–20	−7.9	−55.5	15,769	0.25	10	04.08.2016
13	0–5	−6.0	−48.6	42,262	0.59	51	04.08.2016
14	5–10	−5.4	−42.9	28,135	0.32	12	04.08.2016
15	10–15	−6.8	−49.4	15,511	0.21	5	04.08.2016
16	15–20	−7.5	−53.8	5,717	0.28	10	04.08.2016
17	0–5	−5.2	−42.7	41,366	0.67	80	26.07.2016
18	5–10	−5.5	−45.0	26,940	0.52	49	26.07.2016
19	10–15	−6.6	−49.9	11,915	0.36	24	26.07.2016
20	15–20	−7.1	−50.4	2,085	0.16	5	26.07.2016
21	0–5	−5.9	−43.7	18,398	0.16	11	26.07.2016
22	5–10	−6.3	−49.3	15,386	0.12	10	26.07.2016
23	10−15	−7.9	−56.0	29,312	0.35	51	26.07.2016
24	15−20	−8.4	−56.1	21,288	0.26	20	26.07.2016

### Isotope analysis

The stable isotopes of water in the pore space of the soil samples and the sparkling water samples were analyzed by the direct equilibration method.^9^ Dry air was added to all samples in the airtight bags after which the bags were heat‐sealed and stored for 2 days under constant temperature to allow for an equilibration between the (soil) water sample and the headspace in the bag. Along with the soil and sparkling water samples, standard waters of known isotopic composition were prepared in the same way to derive, via calibration from water vapor isotope data, the liquid‐phase isotopic composition relative to the Vienna Standard Mean Ocean Water. The standard waters used for the calibration were seawater (δ^18^O = –0.85 ± 0.1 ‰ and δ^2^H = –5.1 ± 0.4 ‰, *n* = 4), Aberdeen tap water (δ^18^O = –8.59 ± 0.1 ‰ and δ^2^H = –57.7 ± 0.4 ‰, *n* = 4), and condensate of distilled tap water (δ^18^O = –11.28 ± 0.1‰ and δ^2^H = –71.8 ± 0.4 ‰, *n* = 4). The liquid water isotopic composition of these standard waters was determined with the above‐mentioned OA‐ICOS analyzer running in liquid mode. The seawater was freed from salt by distillation with a rotary evaporator to exclude the effect of salt on the direct equilibration method. The prepared standard waters were analyzed at the beginning, in the middle, and at the end of each day of isotope analysis with the direct equilibration method. We assessed the precision of the analyses from the variation of the three measurements for each of the three standard waters over 29 days of analyses during the last year (261 standard water analyses in total). The average standard deviation for the isotope analysis with the direct equilibration method was found to be 0.54 ‰ for δ^18^O values and 1.39 ‰ for δ^2^H values.

After the 2 days of equilibration at 23 ± 1°C, all the samples of the soil water experiment and the sparkling water experiment were analyzed subsequently by sampling the headspace in the bags with a needle, while a silicone seal outside the bags served as a septum to prevent laboratory air from entering the bag. A tube was connected to the needle to directly route the vapor to the OA‐ICOS instrument running in vapor mode. No carrier gas is needed for this instrument running in vapor mode, since the headspace from the sample bags is directly sucked into the cavity and not diluted. The continuous measurements of δ^18^O and δ^2^H values at 1 Hz were performed for 6 min per sample, and a plateau of stable values for the water vapor concentration in the cavity [ppm], the δ^18^O values [‰], and the δ^2^H values [‰] was reached within 3 min. We averaged the values for δ^18^O and δ^2^H over the last 2 min of the 6 min of the continuous measurements to represent the isotopic composition of the sample. The standard deviation over this last 2 min of integration time after allowing for 4 min to stabilize was always <0.25 ‰ for δ^18^O values and <0.55 ‰ for δ^2^H values. The water vapor concentration at the plateau of the isotope analysis was about 32,000 ppm and its standard deviation over the last 2 min that were used to derive the isotope values was always <90 ppm. To prevent carry‐over effects between different samples, the water vapor concentration in the cavity was reduced to <200 ppm with dry air before each individual analysis.

The TWIA‐45‐EP triple water‐vapor isotope analyzer saves time series of various parameters in addition to the above introduced water vapor concentration and the stable isotopes of water. One dimensionless parameter is called ‘H2Ob_10_PT_B’ and relates to the width of the absorption peaks (R. Provencal, Los Gatos Research, personal communication).

### CO_2_ analysis, GWC, and LOI

Directly after the stable isotope analysis – and, thus, after 2 days of equilibration in the sealed bag with dry headspace – the headspaces of all the soil and sparkling water samples were analyzed for their CO_2_ concentration by gas chromatography (GC) with a flame ionization detector (CP‐9001, Chrompack, Raritan, NJ, USA). A Porapak QS column was used for the GC and the packing was Hayesep Q (60–80 mesh, 2.0 m × 1.8” × 2 mm SS). For the analysis of the headspace from the sample bags, 0.5 mL of the sample vapor was manually directly injected straight onto the column with a syringe. Prior to the CO_2_ analyses of the headspace of the sparkling water and soil sample bags, a calibration was conducted to relate the area under a GC peak to known CO_2_ concentrations. To do so, 0.5 mL of 350, 1000, 3000, 5000, and 10000 ppm concentrations was each injected three times and an average of the resulting area under the GC peak was calculated for each concentration. The resulting linear relationship (*r* = 0.99, *p* < 0.1) was then used to infer the CO_2_ concentration from the area under the GC peak via linear regression. The precision of the CO_*2*_ analyses was estimated from the average standard deviation of the area below the GC peak for the three injections of each of the five different standards of known CO_2_ concentration used for the calibration. The precision for the analyses of the sparkling water samples (Table 1) and the soil samples 1–16 (Table 2) was 94 ppm and for the soil samples 17–24 (measured on a different day) it was 49 ppm. Since the CO_2_ concentrations in the soil and sparkling water samples exceeded the maximum measurement range of the instrument (10,000 ppm), all samples were diluted 1:12 with helium.

The gravimetric water content (GWC) was estimated by oven drying at 105°C overnight and relating the water loss to the field‐moist soil mass. The loss on ignition (LOI) was determined by igniting about 10 g of the previously oven‐dried soil in a muffle‐furnace at 550°C over 2 h and relating the weight loss to the initial dried soil sample.

To statistically test our data for linear relationships, we applied the Pearson correlation (scipy.stats.pearsonr in Python) for the sparkling water experiment, since the data was normally distributed according to the Shapiro–Wilk test (scipy.stats.shapiro in Python) (p‐values for the Shapiro–Wilk test for CO_2_ (*p* = 0.61), δ^18^O (*p* = 0.41), δ^2^H (*p* = 0.41), and the width of the absorption peak (*p* = 0.75). We applied the Spearman rank‐order correlation (scipy.stats.spearmanr in Python) for the soil water experiment, since not all data was normally distributed according to the Shapiro–Wilk test (p‐values for GWC (*p* = 0.02), LOI (*p* < 0.01), width of the absorption peak (*p* = 0.11), and CO_2_ (*p* = 0.52)).

## Results and Discussion

### Does CO_2_ alter the stable isotope analysis with the direct equilibration method?

The experimental set up of applying the direct equilibration method to different volumes of sparkling waters of known isotopic composition allowed us to directly assess the influence of different CO_2_ concentrations in the headspace on the stable isotope analysis with OA‐ICOS, since higher volumes of sparkling water emitted more CO_2_ during the equilibration period (Fig. 1).

**Figure 2:**
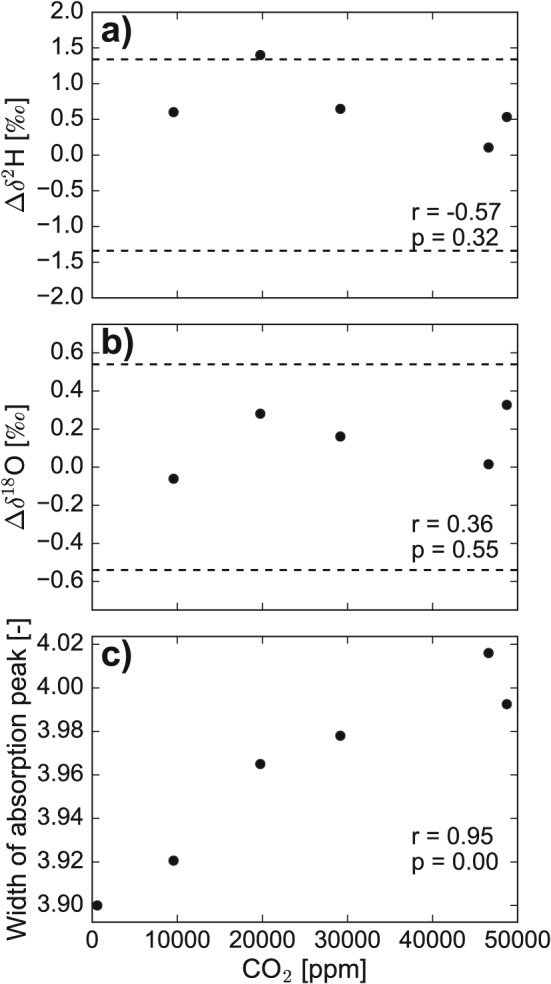
Difference between the measured values for (a) δ^2^H and (b) δ^18^O for non‐sparkling and sparkling waters as a function of the CO_2_ concentration in the sampled headspace. The dashed lines indicate the measurement precision for the applied direct‐equilibration method: 0.54 ‰ for δ^18^O values and 1.39 ‰ for δ^2^H values. (c) Relationship between width of the absorption peak (given as ‘H2Ob_10_PT_B’ from the isotope analyzer) and the CO_2_ concentration in the sampled headspace. The Pearson correlation coefficient is given as r and the significance level is given as p.

The differences in the analyzed stable isotope composition of the non‐sparkling water and the five sparkling water samples of increasing volume leading to increasing CO_2_ concentrations did not show a statistically significant relationship (Figs. 2(a) and 2(b)).

The differences between the measured δ^2^H and δ^18^O values in the headspace of the non‐sparkling water and those of the sparkling waters of various amounts (Δδ) was within the given accuracy range of the instrument for the direct equilibration method over the entire range between 10,000 and 50,000 ppm of CO_2_ (dotted lines in Figs. 2(a) and 2(b)). Consequently, there was also no relationship between the CO_2_ concentration and the lc‐excess.

However, there was a significant positive correlation between the parameter representing the width of the absorption peaks (‘H2Ob_10_PT_B’) and the measured CO_2_ in the headspace (Fig. 2(c)). This relationship shows that CO_2_ in the cavity affects the measurements of the absorption spectra by widening the absorption peaks. However, the integrated area below the spectra is independent of that widening as long as the water vapor concentration in the cavity is constant (D. S. Baer, Los Gatos Research, personal communication). Since the water vapor concentration during the integration period of the isotope measurements did not vary between the samples, there was no effect of CO_2_ on the measured isotope ratios.

As no relationship between the CO_2_ concentration of vapor in the headspace and the stable isotopic composition of the headspace was observed, we have to reject the hypothesis that CO_2_ in the headspace alters the stable isotope analysis results obtained with the direct equilibration method when conducted with an OA‐ICOS instrument. Consequently, in contrast to CRDS, no post‐correction of the sampled isotope data is required for the analysis by OA‐ICOS. However, this finding will be limited to this specific OA‐ICOS laser technology, where the spectral interference does not influence the measurements of isotope ratios. Whether the presence of CO_2_ influences other laser spectroscopy techniques such as Fourier transform infrared (FTIR) or quantum cascade laser (QCL) has not yet been tested.

### Do soil samples emit CO_2_ during storage in the lab?

**Figure 3:**
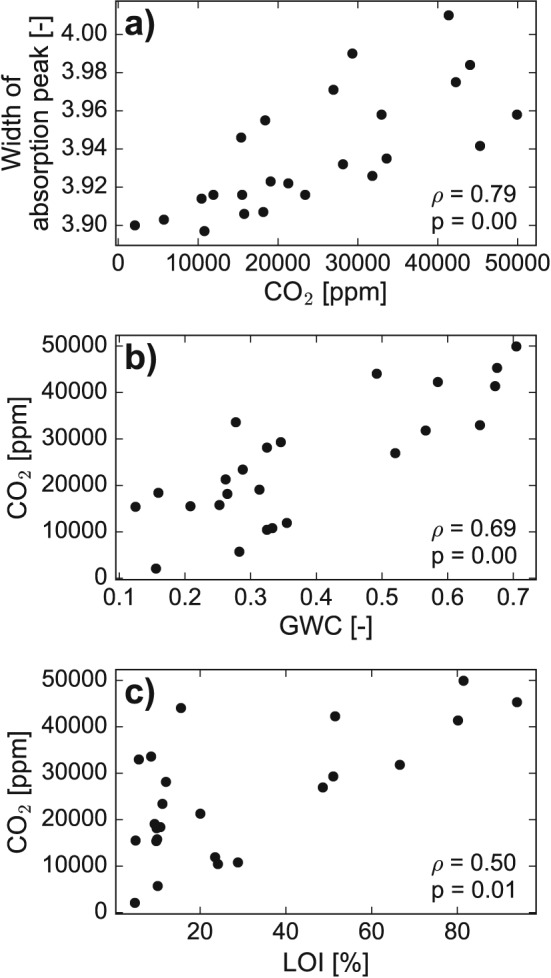
Relationship between (a) the width of the absorption peak (given as ‘H2Ob_10_PT_B’ by the isotope analyzer) and the CO_2_ concentration in the headspace during the stable isotope analysis, (b) CO_2_ concentration and the gravimetric water content (GWC), and (c) CO_2_ concentration and the loss on ignition (LOI). The Spearman rank‐order correlation coefficient is given as ρ and the significance level is given as p.

Similar to the sparkling water experiment, the soil water experiment also revealed that there is a relationship between the width of the absorption peak measured with OA‐ICOS and the CO_2_ concentration in the sampled headspace above the soil sample (Fig. 3(a)). Thus, CO_2_ was emitted from the soil samples during the 2 days of equilibration prior to the isotope analysis. The CO_2_ concentrations varied between 2000 and almost 50,000 ppm (Table 2) and are, therefore, within the range of CO_2_ concentrations covered by the sparkling water experiment.

The CO_2_ concentrations correlate with the soil sampling depth (ρ = 0.74, *p* < 0.01) with a decrease in CO_2_ over soil depth. The CO_2_ concentration also correlates strongly with the GWC (Fig. 3(c)) and less strongly – but still significantly – with the LOI (Fig. 3(d)). A positive relationship between CO_2_ emissions and soil moisture has also been found in studies in the tropics,^29^ in semiarid temperate steppes,^30^ savanna,^24^ and lab experiments.^24^ The LOI is probably a too broad measure of the labile soil organic matter available for microbial activity, since studies on peaty soils have shown that the quality of the organic matter plays an important role for decomposition rates in peaty soils.^26^

We cannot reject the hypothesis that CO_2_ is being generated in bags with soil samples during the 2 days of equilibration time prior to the isotope analysis. Instead, we see high concentrations of CO_2_ and can relate their variability within the soil profile to soil moisture and to some degree also to the amount of organic material in the soil.

### Implications for applications

For the presented soil samples, the lc‐excess values correlate with soil depth (ρ = –0.79, *p* < 0.01) with lower lc‐excess values in the shallow soil and lc‐excess approaching zero at 15–20 cm soil depth. As shown with the sparkling water experiment, for the OA‐ICOS instrument used, the CO_2_ concentration does not affect the isotope analysis. Therefore, we interpret the lc‐excess pattern as kinetic fractionation of the soil water due to soil evaporation under non‐equilibrium processes. This fractionation is more pronounced in the shallow soil that has a more intense interaction with the atmosphere than at 15–20 cm soil depth, where humidity is higher and evaporative fluxes will be lower under the given climatic conditions of the study site. Consequently, the soil water isotopes plot along an evaporation line of slope 4.17. The decrease in the evaporation fractionation signal with depth is a general pattern shown in numerous previous studies.^31^ For soils in temperate forests and temperate grasslands, the evaporation fractionation is usually limited to the upper 30 cm.^31^

Given the high CO_2_ concentrations in the headspace above the soil samples, isotope analysis by WS‐CRDS would require a post‐analysis correction in order to prevent a misinterpretation of a more pronounced soil evaporation signal.

## Conclusions

We conclude that CO_2_ in vapor samples does not affect the measurement of the stable isotopic composition of the water vapor by off‐axis integrated cavity output spectroscopy (OA‐ICOS). Therefore, no post‐correction is needed when applying the direct equilibration method with OA‐ICOS to determine the isotopic composition of soil water. However, as soil samples were shown to emit CO_2_ into the headspace during the 2 days of equilibration prior to the isotope analysis, issues could arise when other laser spectrometry techniques, that are more sensitive to spectral interferences, are applied.

Furthermore, we conclude for the pore water isotopic composition of the soil samples from organic rich peaty podzols that the deviation from the LMWL does not result from spectral interferences between CO_2_ and the isotopologues of water during the analysis. Instead, the location of the soil water isotope samples in the dual isotope plot indicates kinetic fractionation processes that result from evaporation.^31^, ^32^, ^33^ The fact that the evaporation signal, given as the lc‐excess in this study, gets lower with increasing soil depth strongly supports the above interpretation and is in agreement with numerous stable isotope studies that applied various analysis methods.^31^
